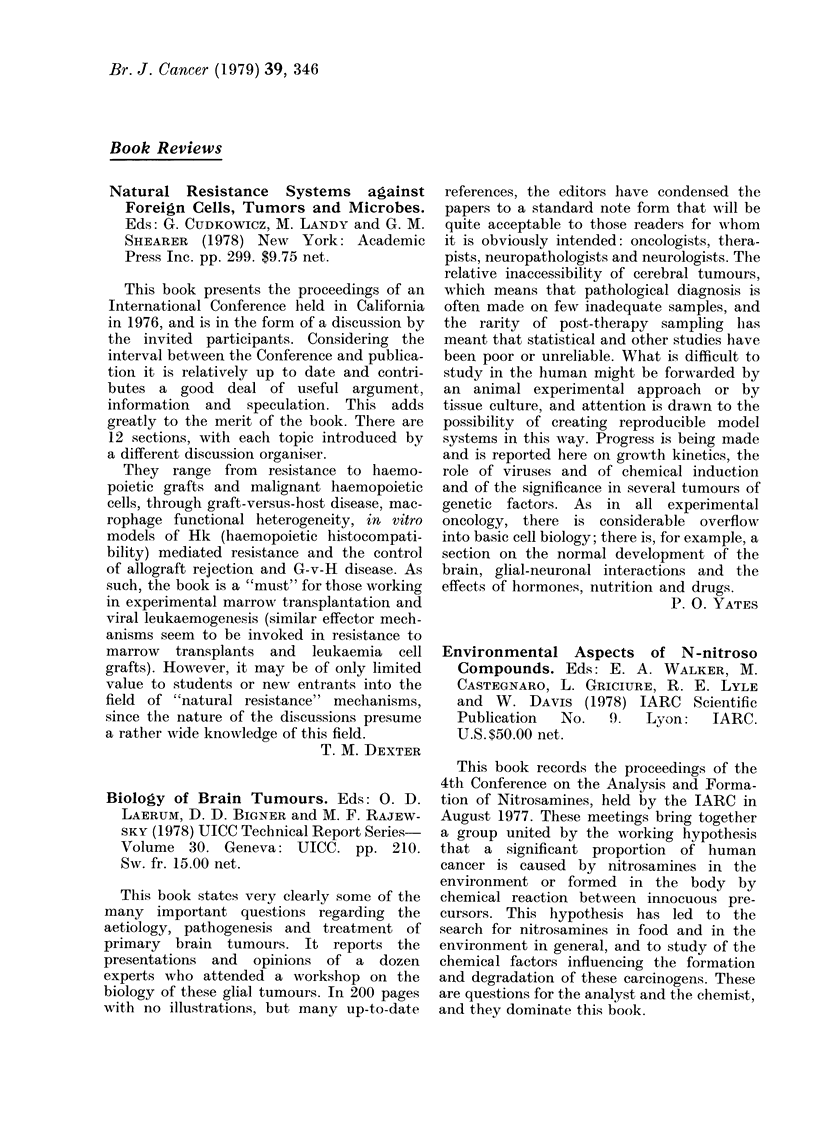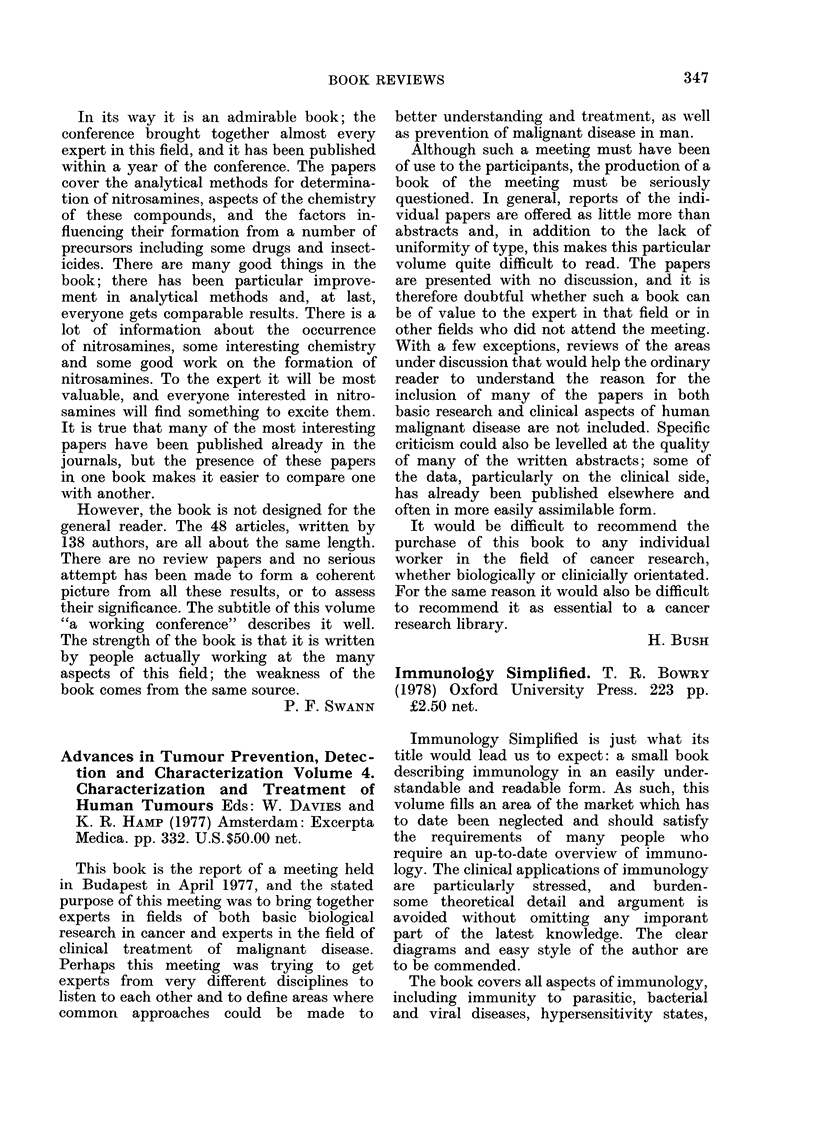# Environmental Aspects of N-nitroso Compounds

**Published:** 1979-03

**Authors:** P. F. Swann


**Environmental Aspects of N-nitroso Compounds.** Eds: E. A. Walker, M. Castegnaro, L. Griciure, R. E. Lyle and W. Davis (1978) IARC Scientific Publication No. 9. Lyon: IARC. U.S.$50.00 net.

This book records the proceedings of the 4th Conference on the Analysis and Formation of Nitrosamines, held by the IARC in August 1977. These meetings bring together a group united by the working hypothesis that a significant proportion of human cancer is caused by nitrosamines in the environment or formed in the body by chemical reaction between innocuous precursors. This hypothesis has led to the search for nitrosamines in food and in the environment in general, and to study of the chemical factors influencing the formation and degradation of these carcinogens. These are questions for the analyst and the chemist, and they dominate this book.

In its way it is an admirable book; the conference brought together almost every expert in this field, and it has been published within a year of the conference. The papers cover the analytical methods for determination of nitrosamines, aspects of the chemistry of these compounds, and the factors influencing their formation from a number of precursors including some drugs and insecticides. There are many good things in the book; there has been particular improvement in analytical methods and, at last, everyone gets comparable results. There is a lot of information about the occurrence of nitrosamines, some interesting chemistry and some good work on the formation of nitrosamines. To the expert it will be most valuable, and everyone interested in nitrosamines will find something to excite them. It is true that many of the most interesting papers have been published already in the journals, but the presence of these papers in one book makes it easier to compare one with another.

However, the book is not designed for the general reader. The 48 articles, written by 138 authors, are all about the same length. There are no review papers and no serious attempt has been made to form a coherent picture from all these results, or to assess their significance. The subtitle of this volume "a working conference" describes it well. The strength of the book is that it is written by people actually working at the many aspects of this field; the weakness of the book comes from the same source.

P. F. Swann